# Coverage recommendation for genotyping analysis of highly heterologous species using next-generation sequencing technology

**DOI:** 10.1038/srep35736

**Published:** 2016-10-20

**Authors:** Kai Song, Li Li, Guofan Zhang

**Affiliations:** 1 Key Laboratory of Experimental Marine Biology, Institute of Oceanology, Chinese Academy of Sciences, Qingdao, Shandong, 266071, China; 2 Laboratory for Marine Fisheries and Aquaculture, Qingdao National Laboratory for Marine Science and Technology, Qingdao, Shandong, 266071, China; 3 National & Local Joint Engineering Laboratory of Ecological Mariculture, Institute of Oceanology, Chinese Academy of Sciences, Qingdao, Shandong, 266071, China; 4 Laboratory for Marine Biology and Biotechnology, Qingdao National Laboratory for Marine Science and Technology, Qingdao, Shandong, 266071, China

## Abstract

Next-generation sequencing (NGS) technology is being applied to an increasing number of non-model species and has been used as the primary approach for accurate genotyping in genetic and evolutionary studies. However, inferring genotypes from sequencing data is challenging, particularly for organisms with a high degree of heterozygosity. This is because genotype calls from sequencing data are often inaccurate due to low sequencing coverage, and if this is not accounted for, genotype uncertainty can lead to serious bias in downstream analyses, such as quantitative trait locus mapping and genome-wide association studies. Here, we used high-coverage reference data sets from *Crassostrea gigas* to simulate sequencing data with different coverage, and we evaluate the influence of genotype calling rate and accuracy as a function of coverage. Having initially identified the appropriate parameter settings for filtering to ensure genotype accuracy, we used two different single-nucleotide polymorphism (SNP) calling pipelines, single-sample and multi-sample. We found that a coverage of 15× was suitable for obtaining sufficient numbers of SNPs with high accuracy. Our work provides guidelines for the selection of sequence coverage when using NGS to investigate species with a high degree of heterozygosity and rapid decay of linkage disequilibrium.

Next-generation sequencing (NGS) technology is a powerful and cost-effective approach for large-scale DNA sequencing^1^. The arrival of NGS technologies in the marketplace has changed the way we think about scientific approaches in genetic and evolutionary research. The major advance offered by NGS is the ability to produce an enormous volume of data cheaply—in some cases in excess of one billion short reads per instrument run. Currently, NGS technologies are being applied to an increasing number of non-model animals and plants, including. cattle^2^, pigs^3^,^4^, rice^5^,^6^, maize^7^, soybean^8^, and cucumber^9^, to reveal the genetic basis of economic traits, local adaptation, and evolution in extreme environments.

For numerous non-model organisms, there exist genomic resources that can provide an important basis for the application of NGS technology. Through large-scale sequencing, it is not difficult to obtain NGS short-read data for these organisms. Analysis pipelines that deal with these NGS data include two steps, read mapping and genotype calling. The bioinformatics software related to these two steps are fully and widely used. Most read mapping algorithms for NGS data are based on either ‘hashing’ or an effective data compression algorithm called the ‘Burrows–Wheeler transform’ (BWT). The BWT-based aligners include, BWA^10^, Bowtie2^11^, and SOAP2^12^, which are all fast, memory-efficient, and particularly useful for aligning repetitive reads. Genotype calling, the analysis subsequent to read mapping, aims to convert base calls and quality scores into a set of genotypes for each individual in a sample. The two widely used software for genotype calling are SAMtools^13^ and GATK^14^.

In research using NGS technologies, the most important consideration is to obtain single-nucleotide polymorphism (SNP) sites with accurate genotypes. For genome-wide association or QTL mapping studies for economic traits, in particular, the accuracy of genotyping SNP sites has a significant influence on the results obtained. If only a small fraction of SNP sites are incorrectly genotyped, this could lead to radically different conclusions. In order to obtain accurate genotypes for each individual in a sample, higher coverage sequencing depth for each individual is a possible solution. Twenty times or even higher coverage is possibly sufficient for accurate genotyping. However, for some non-model organisms, higher coverage sequencing for a large population would be expensive and beyond the means of many researchers. Another approach, low-coverage sequencing assisted by genotype imputation methods, is a possible solution for population studies of some organisms. This method has been very successfully applied to association studies of humans^15^ and rice^5^,^6^. In particular, in association studies of rice agronomic traits, researchers used onefold-coverage genome sequencing per individual and the kNN (k-nearest neighbour algorithm) based on the imputation method to accurately determine genotypes and perform genome-wide association studies^5^.

However, the aforementioned methods have not been successfully applied to a large group of organisms with a high degree of heterozygosity and rapid decline in linkage disequilibrium (LD). This is because, compared with the low-coverage sequencing for an inbred line of rice, which could be considered as a diploid, it is very difficult to identify the genotypes for heterozygous SNP sites with very low covered reads. Moreover, genotype inference methods, which are based on the genotypes of contiguous SNP sites, may be problematic because of typing errors for heterozygous sites and lower linkage disequilibrium. Thus, genotyping based on higher sequencing depth is essential for organisms that have a high degree of heterozygosity and low LD values. The choice of appropriate sequencing coverage is accordingly an important consideration in the study of such organisms using NGS technology.

The Pacific oyster, *Crassostrea gigas*, is a marine bivalve belonging to the phylum Mollusca, which contains the largest number of described marine animal species. Oysters are important fishery and aquaculture species, as well as being models for studying neurobiology, biomineralisation, ocean acidification, and adaptation to coastal environments under climate change^16^,^17^. The genome of *C. gigas*, which was first published in 2012, provides a basic resource for genetic and evolutionary studies^18^. *C. gigas* has a modest genome size of less than 500 million bp (Mbp), a short generation time (1 year), and worldwide distribution, which makes it a suitable model molluscan species. Furthermore, the polymorphism rate for *C. gigas* is 1.3% and the linkage disequilibrium decays to less than 0.2 in approximately 20 bps, which makes *C. gigas* a suitable representative organism with high diversity and a rapid decay of LD for the study of sequencing coverage choice^18^.

In this study, we used simulations to investigate genotyping accuracy as a function of sequence coverage and other filter parameters based on NGS sequencing data for *C. gigas*. Using two different SNP calling pipelines, we found that a coverage of 15× was suitable for NGS analysis to obtain a sufficient number of SNPs called with high accuracy. On the basis of our findings, we provide guidelines for the selection of sequencing coverage for NGS application to organisms with a high degree of heterozygosity.

## Results

To investigate genotype calling rate and accuracy using high-throughput sequencing data as a function of sequencing coverage in *C. gigas*, we used three wild samples collected from Weifang, Rongcheng, and Lianyungang (three Chinese coastal cities). Sequencing of these samples was performed using the HiSeq 2000 platform (Illumina) according to manufacturer’s standard protocols. We mapped the DNA sequence reads of the three samples to the oyster genome by using the software BWA. After read mapping and subsequent strict filtering, the average depth was approximately 20× for all three samples (Table 1). To obtain high-quality SNPs, SAMtools was selected for SNP calling using both single-sample and multi-sample pipelines. In the subsequent analysis, to evaluate the calling rate and accuracy of genotyping as a function of sequencing coverage, we simulated the sequencing data of different coverages by sampling the mapped reads without replacement.

### The effect of read depth on genotype calling

**Figure 1:**
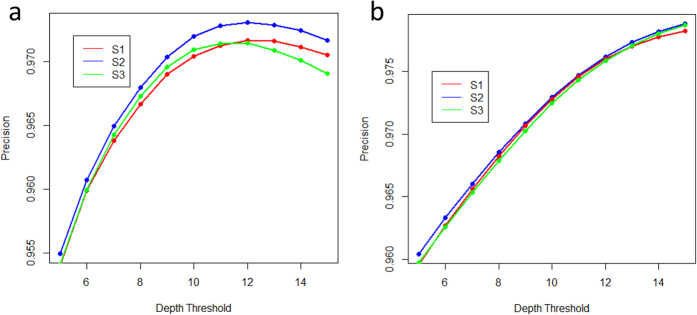
The precision of the SNP genotyping under different read depth threshold. The genotyping accuracy for SNPs under different DP parameters, from 5× to 15×, using the sampled data with coverage 5× (**a**) and 10× (**b**) for each individual. S1, S2 and S3 represent the three samples used in the study.

When analysing sequencing data, researchers often use strict filters to account for the uncertainty associated with genotype calls. Genotype quality (GQ) and read depth (DP) are two commonly used filter parameters for determining the genotype for each SNP site. In this study, we fixed the parameter of GQ threshold as 20 and then evaluate the influence of different DP on the accuracy of SNP genotyping. Firstly, we chose SNP sites that had more than 20 reads covered in each sample as the benchmark SNP sets. We assumed that because these SNPs had sufficient read depth, the genotype inference for them should be accurate. Using the sampled data with coverage 5× and 10× for each individual, we then investigated the genotyping accuracy for the SNPs as a function of the DP parameter. The results showed that the accuracy was greater than 95% when the DP threshold was 5 and could reach 97% when DP threshold was larger than 10 (Fig. 1a,b). Accordingly, we reasoned that in order to determine genotypes with sufficient accuracy, the GQ parameter should be set to 20, and that this should be used in conjunction with a DP threshold of 10. In the subsequent analysis, we filtered the genotypes using the parameter thresholds of GQ ≥20 and DP ≥10.

### The effect of coverage on single-sample calling

In order to evaluate the genotype calling rate and accuracy under different coverage, we simulated the sequencing data using the sampling method and then called the SNPs for each subgroup of sequencing data. Initially, we filtered the SNP sets obtained from the total sequencing data using the parameters GQ and DP (GQ ≥20 and DP ≥10). We then used these filtered SNPs as benchmarks because the genotype accuracy for them was high. We accordingly identified 3,909,589, 3,745,142, and 3,754,426 high quality SNPs for the three *C. gigas* samples, respectively.

**Figure 2:**
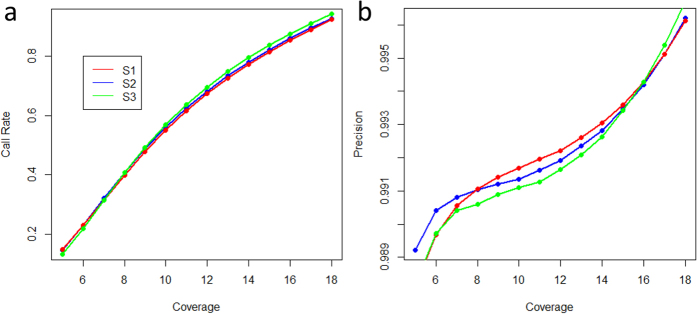
The calling rate and genotype accuracy under different sequence coverage. (**a**) The calling rate of genotypes for the three samples using the single sample pipeline under different coverage. (**b**) The precision of genotypes called for the three samples using the single sample pipeline under different coverage.

We subsequently investigated the calling rate and genotype accuracy as a function of coverage. We observed that the fraction of called SNPs increased sharply as the coverage increased from 5× to 18× (Fig. 2a). The fraction of SNPs obtained was below 20% when the coverage was 5×, but was greater than 80% when the coverage was greater than 15×. Furthermore, the precision of the called SNPs was very high. When the coverage was larger than 7×, the precision could be greater than 99% (Fig. 2b).

On the basis of the aforementioned results, we considered that a coverage of 10× or 15× would be suitable for individual SNP calling analysis for an organism with very high diversity. When the coverage was 10×, approximately 55% of the total SNPs could be called, whereas with a coverage of 15×, the fraction could be greater than 80%. Moreover, the genotype accuracy of the SNPs obtained using the two different coverages could be very high (>99%).

### The effect of coverage on multi-sample calling

**Figure 3:**
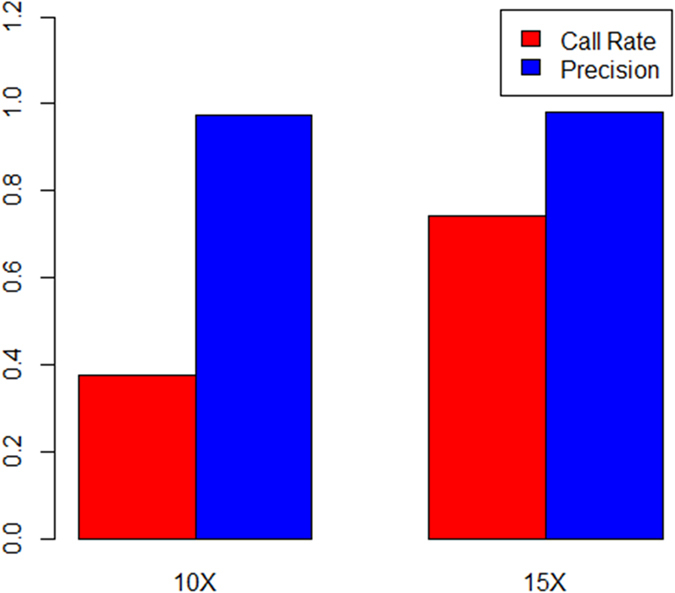
The calling rate and genotype accuracy using multi-sample pipeline. The calling rate and genotype accuracy under the two different coverage, 10× and 15×, using multi-sample pipeline.

When using the multi-sample calling method, we used the sequencing data of all three *C. gigas* samples as the input for SAMtools, and called the SNP sites simultaneously. We also focused on the calling rate and accuracy of genotypes under different sequencing coverage. As benchmarks, we used only SNP sites for which genotypes were determined in all three samples (using the following parameters: GQ ≥20 and DP ≥10). In total, 5,022,415 SNP sites were found to be polymorphic in the three samples. We then investigated the calling rate and accuracy under two different levels of coverage, 10× and 15×. The results showed that the calling rate and accuracy were both a little lower than those obtained using the single-sample calling pipeline. Under 10× coverage, the calling rate was very low, with only approximately 37.4% of the total SNP sites being identified from the three samples. The accuracy of the genotypes determined was 97.2%. When the coverage was 15×, the calling rate could reach 75% and the genotype accuracy was greater than 98% (Fig. 3). Accordingly, we considered that 15× was a suitable coverage for accurately genotyping a sufficient number of SNPs.

## Discussion

In this study, we evaluated the effect of sequencing coverage on SNP calling rate and genotyping accuracy. Initially, we examined how the SNP filter parameters influence the genotype accuracy, and thereby determined appropriate parameter settings. Then, by simulating the sampling processes, we evaluated the genotype calling rate and accuracy under different sequencing coverage. Using two different SNP calling pipelines, we found that a coverage of 15× was suitable for genotyping a sufficient number of SNPs with high accuracy. The fractions of SNPs obtained under the two different calling pipelines, single-sample and multi-sample, were both very high (greater than 75%). Thus, we considered that a coverage of 15× was a suitable choice for obtaining a sufficient number of accurately genotyped SNPs in an organism with a very high number of heterozygous sites.

Oysters belong to the class of bivalve molluscs, which has one of the highest diversities among animal phyla^19^. One of the distinguishing features of *C. gigas* is that it has a high degree of heterozygosity for polymorphic sites. We found almost 60% of the SNPs in *C. gigas* are heterozygous, which made the inference of genotypes very difficult. When the sequencing coverage was low, heterozygous sites are very likely to be inferred as homozygous. Moreover, such mistakes are very difficult to correct based on the sequencing data. Other widely used approaches for genotyping are the imputation-based methods^5^,^15^,^20^,^21^,^22^,^23^,^24^. These methods have been fully developed and successfully applied in studies of model organisms, including humans, *Drosophila*, and rice. Imputation-based methods take advantage of the pattern of LD at nearby sites to infer genotypes, and as a result, genotype calling accuracy is significantly improved and missing genotypes can be imputed. However, in oysters, the genotypes of nearby sites inferred from low coverage sequencing are not convincing, and the pattern of LD decayed rapidly, both of which make genotype imputation based on low coverage sequencing very difficult.

**Figure 4:**
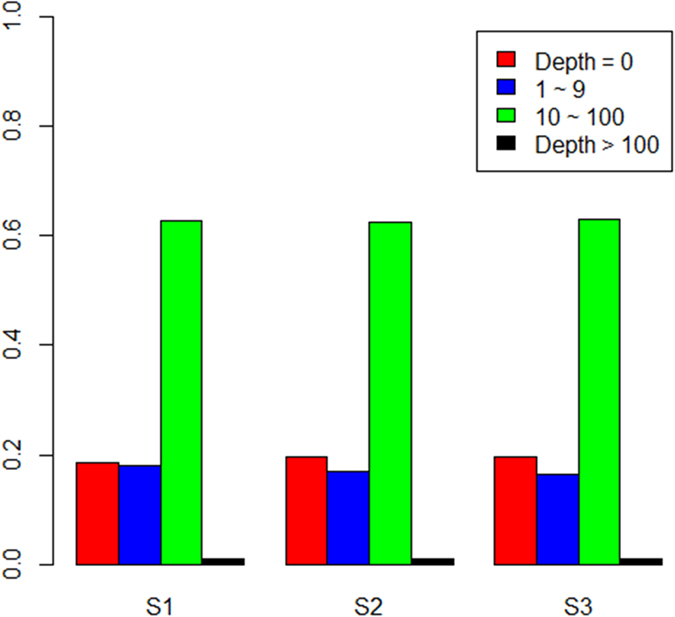
The fraction of the genome under different depth. We grouped the depth as four categories: no reads covered (depth = 0), 1~9, 10~100, and more than 100.

One of the factors that influence the performance of single- and multi-sample calling pipelines is the non-uniform distribution of sequencing reads across a genome. We found that approximately 40% of the genome regions were covered less than 10× using the total sequencing data for each sample. Notably, approximately 20% of the regions did not have a single covered sequence read (Fig. 4). We checked whether these regions were indeed covered by reads or not. We retained those reads that were filtered (not uniquely mapped or mismatch nucleotides larger than five) and found the regions these reads covered had approximately 35% overlap with the regions covered by no reads. Consequently, at the whole-genome level, approximately 13% of the total region was truly not covered by the sequencing data. We considered that there are perhaps two reasons that could explain this: one is that these regions are highly repetitive regions, the other is that these regions had high diversity, making read mapping difficult.

**Figure 5:**
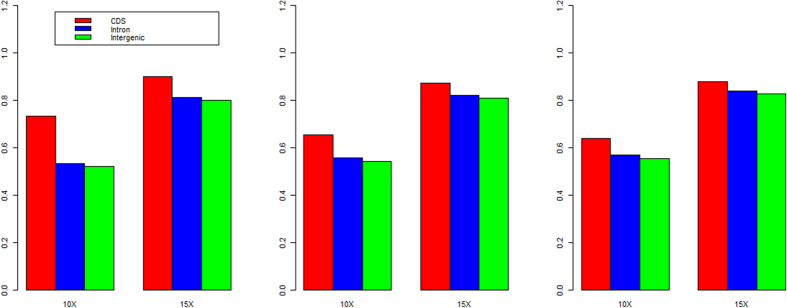
The genotype calling rate in different genomic functional regions. (**a–c**) We tested the genotype calling rate under the two different coverage, 10× and 15×, in the three genomic functional regions, coding, intron and intergenic regions for the three samples using single sample pipeline.

**Figure 6:**
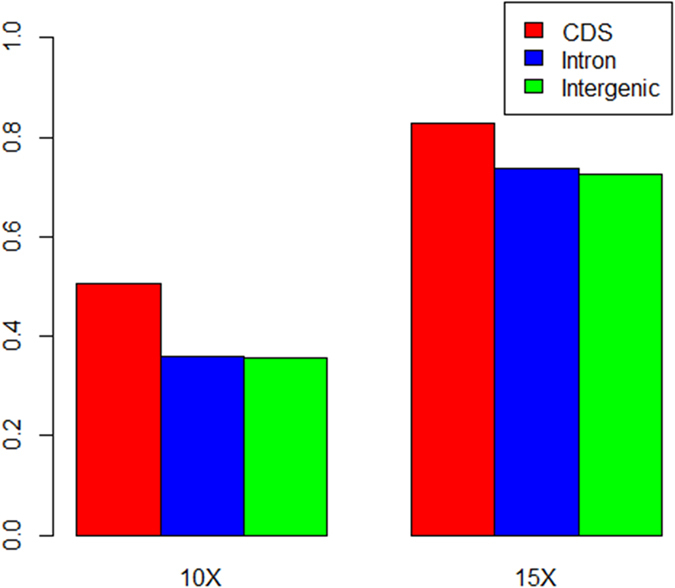
The genotype calling rate in different genomic functional regions using multi-samples pipeline. We tested the genotype calling rate under the two different coverage, 10× and 15×, in the three genomic functional regions, coding, intron and intergenic regions for the three samples using multi-samples pipeline.

We also checked whether the genotype calling rates were different in different functional regions in the oyster genome. We found that calling rate was highest in the coding regions and lowest in the intergenic regions. The single sample calling rate of coding regions could reach approximately 70% when the sequencing coverage was 10×, and greater than 85% when coverage was 15× (Fig. 5). Using multi-sample calling pipeline analysis, the calling rate in the coding regions could be greater than 50% when the coverage was 10×, and greater than 80% when the coverage was 15× (Fig. 6). The above results indicate that the complexity of the genome indeed influences the genotype calling rate. The intergenic regions had greater diversity than the coding and intron regions, which made mapping of the sequenced reads very difficult. Consequently, the SNP calling in these regions was also reduced.

In order to investigate what the genotyping calling rate and accuracy would be when the sequencing coverage was very high; we used the sequencing data in the work of the oyster genome^18^. After read mapping and strict filtering, the average depth was about 104×. Then, we found the fraction of called SNPs increase quickly as the coverage increase from 5× to 20× (Supplementary Fig. S1a). The calling rate was more than 60% when the coverage was 15× and was about 73% when the coverage was 20×. Also, we found the precision of the called SNP increased sharply from 5× to 15× and very slowly after 15× (Supplementary Fig. S1b). The combination of calling rate and calling accuracy gives a clear pattern of the relationship between them as the sequencing coverage increasing. The calling rate and calling accuracy both increased quickly from 5× to 15×. The increased calling rate was limit when the coverage was more than 15× (Supplementary Fig. S2). Also, we found the calling rate of coding region was more than 70% when the coverage was 15×. These all suggest that the coverage of 15× was a good choice in consideration of both of the genotype calling rate and sequencing cost.

In this study, we examined how the SNP filter parameters influence the genotype accuracy. The parameters choice suggestions could be used as a reference and be applicated to the genotyping work in other species with high level of heterozygous sites. However, the choice of sequence coverage is complicated and associated with several factors, e.g. the heterozygous sites density along the genome, the LD pattern and the genomic complexity. Thus, the recommended coverage was not suitable to be applied to other species directly. But we thought our study could provide a reference to the investigation in other species.

One may argue that future studies will have increased coverage and that many of the problems highlighted herein will be solved; nevertheless, even with limited funding, we expect to be able to genotype a sufficient number SNP sites with high accuracy under suitable sequencing coverage. The insights gained here suggest how selection of an appropriate coverage for those organisms with high diversity can provide useful basic information for subsequent analyses.

## Materials and Methods

### Samples and genome sequencing

Three wild Pacific oysters, *Crassostrea gigas*, were collected from three Chinese cities, Weifang, Rongcheng, and Lianyungang. Genomic DNA was extracted from mantle tissue using a standard phenol-chloroform method. For each individual, 1–15 μg of DNA was sheared into fragments of 200–800 bp using the Covaris system (Life Technologies). DNA fragments were then treated according to the Illumina DNA sample preparation protocol. Fragments were end-repaired, A-tailed, ligated to paired-end adaptors, and PCR amplified with 300–500 bp inserts for library construction. Sequencing was performed to generate 100-bp paired-end reads using the HiSeq 2000 platform (Illumina) according to the manufacturer’s standard protocols.

### Read mapping and SNP calling

Filtered reads from all individuals were aligned to the oyster reference genome^18^ by the Burrows-Wheeler Aligner (BWA)^10^ using the command ‘aln’ with the default parameters. ‘bwa sampe’ was then used to generate SAM files for each individual. Next, we improved the alignment results using the following three steps: (1) Alignment reads that mapped to more than one position in the genome were filtered; (2) Alignment reads with a mismatch greater than 5 and mapping quality less than 20 were filtered; (3) Remove potential PCR duplication. The remaining mapped reads were regarded as high-quality SAM files.

After alignment, we performed SNP calling for the three samples using a Bayesian approach as implemented in the package SAMtools. The ‘mpileup’ command was used to identify SNPs with the parameters ‘-C 50 -t DP -t SP -ug’. Both the single-sample and multi-sample calling pipelines were used in the study. The SNPs with genotype quality ≥20 and coverage depth ≥10 were retained as the benchmark SNP sets for the subsequent analysis.

### Sampling method

In order to simulate the sequencing data under different coverage, the sampling method we used involved randomly selecting parts of the data in the high-quality SAM files. We generated a series of random numbers to represent the high-quality mapped reads and then sampled from the total mapped reads without replacement. We selected different coverage levels from 5× to 18×, with a 1× step between levels. The sampling processes were repeated five times to get the mean values for the calling rate and precision.

### Filter parameter evaluation

We fixed the genotype quality as 20 and evaluated the genotype accuracy under different parameters of read depth (DP). We used the SNP sites with DP values larger than 20 called from the total high-quality mapped reads as the benchmark SNP sets. We then used two levels of lower coverage (5× and 10×) to evaluate the genotype accuracy under different thresholds of read depth. The depth was set at 5 to 15.

## Additional Information

**How to cite this article**: Song, K. *et al*. Coverage recommendation for genotyping analysis of highly heterologous species using next-generation sequencing technology. *Sci. Rep.* **6**, 35736; doi: 10.1038/srep35736 (2016).

## Supplementary Material

Supplementary Information

## Figures and Tables

**Table 1:** Statistics of NGS data for each sample.

Sample	No. of raw reads	No. of mapped reads after strict filtering	Mapped ratio (%)	Usable depth (X)
S1	203,570,504	108,661,117	53.37	19.8338639
S2	206,222,916	111,823,708	54.22	20.41113019
S3	181,599,328	101,078,316	55.66	20.49975412
