# Supplementary material for: Coverage recommendation for genotyping analysis of highly heterologous species using next-generation sequencing technology
Source: Sci Rep. 2016 Oct 20;6:35736. doi: 10.1038/srep35736 (PMC5071758; doi:10.1038/srep35736)
Supplement: Supplementary Information [file srep35736-s1.pdf]

Supplymentary Information for

Coverage recommendation for genotyping analysis of highly heterologous species using next-generation sequencing technology

Kai Song[1,3,4], Li Li[1,3,4], Guofan Zhang[1,2,4] *

Affiliations and addresses

1. Key Laboratory of Experimental Marine Biology, Institute of Oceanology, Chinese Academy of Sciences, Qingdao, Shandong, China 266071

2. Laboratory for Marine Biology and Biotechnology, Qingdao National Laboratory for Marine Science and Technology, Qingdao, Shandong, China 266071

3. Laboratory for Marine Fisheries and Aquaculture, Qingdao National Laboratory for Marine Science and Technology, Qingdao, Shandong, China 266071

4. National & Local Joint Engineering Laboratory of Ecological Mariculture, Qingdao, Shandong, China 266071

* Corresponding author Email: gzhang@qdio.ac.cn

This file includes:

Supplymentary figures S1-S2

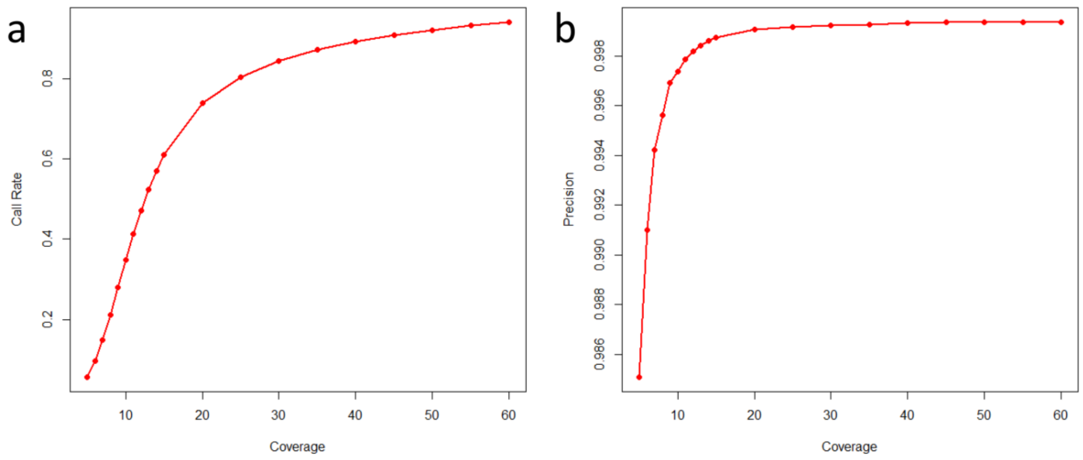

Figure S1. The calling rate and genotype accuracy under different sequence coverage using a extremely high sequencing coverage data. (a), the calling rate of genotypes using the single sample pipeline under the coverage from 5× to 60×. (b), the precision of genotypes using the single sample pipeline under the coverage from 5× to 60×.

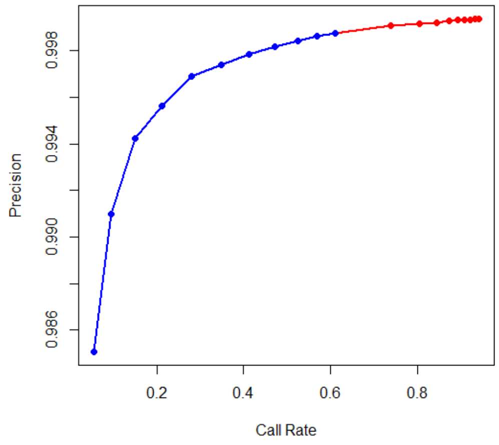

Figure S2. The relationship between calling rate and genotype accuracy under different coverage. The two dimensional plot relating calling rate and genotype accuracy under the coverage from 5× to 60×. The blue points and lines represent the coverage from 5× to 15×, with a 1× step between each point. The red points and lines represent the coverage from 20× to 60×, with a 5× step between each point
